# Genotype-Phenotype Delineation of Autoimmune Polyendocrinopathy, Candidiasis, and Ectodermal Dystrophy in a Pediatric Patient: A Case Report

**DOI:** 10.3390/genes17020160

**Published:** 2026-01-29

**Authors:** Rima Hanna-Wakim, Pascale E. Karam, Mazen Kurban, Nadine Yazbeck

**Affiliations:** 1 Division of Pediatric Infectious Diseases, Department of Pediatrics and Adolescent Medicine, American University of Beirut, Beirut 1107, Lebanon; rh08@aub.edu.lb; 2 Center for Infectious Disease Research, American University of Beirut, Beirut 1107, Lebanon; 3 Inherited Metabolic Diseases Program, Department of Pediatrics and Adolescent Medicine, American University of Beirut, Beirut 1107, Lebanon; pk06@aub.edu.lb; 4 Department of Dermatology, American University of Beirut, Beirut 1107, Lebanon; 5 Division of Pediatric Gastroenterology and Nutrition, Department of Pediatrics and Adolescent Medicine, American University of Beirut, Beirut 1107, Lebanon

**Keywords:** *AIRE*, APECED, hepatitis, chronic mucocutaneous candidiasis, alopecia

## Abstract

Background/Objectives: Autoimmune Polyendocrinopathy with Candidiasis and Ectodermal Dystrophy is an extremely rare autosomal recessive disorder caused by inborn errors of immunity. It is due to a loss-of-function mutation in the *AIRE* autoimmune regulator gene. Its manifestations include autoimmunity affecting endocrine glands, in addition to non-endocrine manifestations including dental enamel hypoplasia, alopecia areata, hepatitis, and chronic mucocutaneous candidiasis. Globally, 10 cases per million are affected by this condition, with higher incidence in highly consanguineous populations. Here, we describe a novel *AIRE* gene mutation in a pediatric patient from Lebanon, along with the observed phenotype. Method: A nine-year-old boy with history of craniosynostosis presented with jaundice. His past medical history was significant for recurrent oral thrush, keratoconjunctivitis, nail dystrophy, and alopecia. Upon presentation, he had jaundice, isolated splenomegaly, and severe failure to thrive. Laboratory tests showed transaminitis, cholestasis, and hypergammaglobulinemia. Abdominal ultrasound findings were suggestive of cirrhosis with compensated portal hypertension. The differential diagnosis included viral infection, inborn errors of metabolism, and autoimmune hepatitis. Results: Exome sequencing identified a novel homozygous pathogenic variant in the *AIRE* gene, NM_000383.4: c.1066dup p.(Arg356Profs*16), confirming the diagnosis. Conclusions: This study expands the genotypic and phenotypic spectrum of a rare inborn error of immunity in a child with chronic mucocutaneous candidiasis, enamel hypoplasia, and hepatitis.

## 1. Introduction

Autoimmune Polyendocrinopathy with Candidiasis and Ectodermal Dystrophy (APECED) (also known as Autoimmune Polyendocrine syndrome Type I (OMIM: 240300) is an autosomal recessive disorder of inborn errors of immunity. It is due to a loss of function mutation in the autoimmune regulator (*AIRE*) gene impairing immune tolerance in the thymus, and results in peripheral escape of self-reactive T lymphocytes and the generation of several cytokines and tissue-antigen targeted autoantibodies [1]. This results in failure of T cell tolerance within the thymus [2]. Over 100 different disease-causing pathogenic variants have been reported [3]. The most common pathogenic variant is the Finnish major mutation p.R257X, prevalent in people in Finland, Russia, and Eastern Europe [4]. Another common pathogenic variant (p.C322del13) is prevalent in persons in France, and North America [3,5]. This extremely rare disorder has an incidence averaging 10 cases per million globally; however, it is more common in populations with high consanguinity rates, such as in Lebanon, where consanguineous marriages remain relatively common [6,7].

The manifestations of APECED include autoimmunity affecting endocrine glands, leading to hypoparathyroidism, hypothyroidism, adrenal insufficiency, diabetes, gonadal dysfunction, and other endocrine abnormalities. Additionally, patients may exhibit non-endocrine manifestations including dental enamel hypoplasia, alopecia areata, enteropathy, hepatitis, pernicious anemia, and chronic mucocutaneous candidiasis [1,8]. In particular, hepatitis can present early, even before the development of classic APECED manifestations [9].

Here, we describe a novel *AIRE* gene mutation in a Lebanese child, presenting with a history of chronic mucocutaneous candidiasis, alopecia, and hepatitis.

## 2. Case Presentation

A 9-year-old boy with a history of craniosynostosis, for which he had undergone multiple corrective surgeries, presented with jaundice at the age of 7 years, following his most recent procedure. Initial imaging of the head showed cloverleaf skull and premature craniosynostosis of the bilateral frontal, lambdoid, and of the sagittal sutures. His clinical history dates to the age of four months with recurrent oral thrush. At the age of three and a half years, he developed nail dystrophy, characterized by his nails “falling off”, along with scalp patches suggestive of alopecia. He was intermittently treated with oral steroids until the age of 7 years. In addition, he had severe gingival inflammation, enamel erosion, and multiple episodes of keratoconjunctivitis.

**Figure 1:**
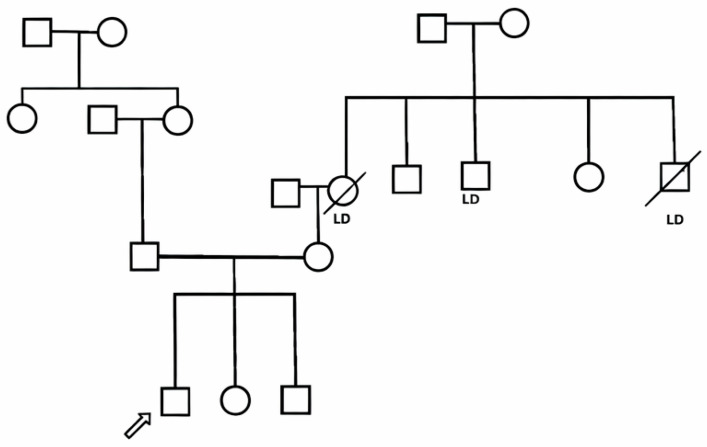
Pedigree of the family of a patient with Autoimmune Polyendocrinopathy with Candidiasis and Ectodermal Dystrophy. Arrow indicates the index patient. LD: subjects with liver disease.

The child had also recurrent episodes of otitis media since infancy and one episode of suspected viral meningitis at 6 years of age. The parents are first-degree cousins, and the family history is notable for liver disease of unknown etiology in three relatives, leading to liver transplantation in the maternal great-uncle and aunt (Figure 1).

**Figure 2:**
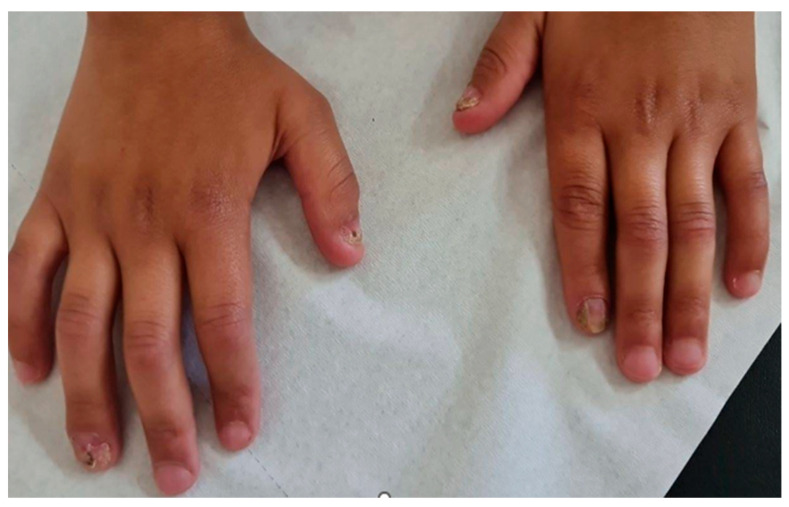
Enamel hypoplasia and dorsal pterygium affecting several fingernails in a patient with Autoimmune Polyendocrinopathy with Candidiasis and Ectodermal Dystrophy.

On presentation, his physical exam was significant for jaundice and severe failure to thrive, with a weight at the 2.84th percentile and height at the 2.16th percentile based on the World Health Organization Child Growth Standards. He had cranial deformity, with diffuse hair rarefaction across the scalp without signs of scarring or peri-follicular erythema. He also had bilateral esotropia, keratitis, enamel hypoplasia, as well as dorsal pterygium over several fingernails, as shown in (Figure 2) (photograph obtained before steroids exposure. No evidence of Kayser–Fleischer rings. On abdominal exam, there was isolated splenomegaly.

Initial laboratory tests showed cholestasis with total bilirubin of 4 mg/dL and direct bilirubin 2.8 mg/dL (normal values for age: total less than 1.2 mg/dL and direct less than 0.3 mg/dL). Alanine aminotransferase (ALT) and aspartate aminotransferase (AST) levels were elevated at 208 IU/L and 181 IU/L, respectively (normal values for age ALT 0–65 IU/L and AST 0–50 IU/L). Serum albumin was low at 26 g/L (normal range: 36–53 g/L). The international normalized ratio was prolonged at 2.1 (normal range: 0.9–1.2). Abdominal ultrasound with Doppler revealed a normal liver size (10 cm; normal for 9–11 years: 7.5–13.5 cm), but with diffuse heterogeneous echotexture and irregular margins, atrophy of the right hepatic lobe, and hypertrophy of the left hepatic lobe, consistent with chronic liver disease and cirrhosis. Additionally, the spleen was enlarged at 14.3 cm in craniocaudal dimension (normal for 9–11 years: 6.5–11 cm). These findings were suggestive of chronic liver disease/cirrhosis with compensated portal hypertension.

The differential diagnosis initially included viral infections, inborn errors of metabolism, and autoimmune hepatitis. Because viral etiologies are common causes of pediatric hepatitis and can both trigger and mimic autoimmune or metabolic liver injury, we systematically excluded these by testing serologies for hepatitis A, B, and C, as well as Epstein–Barr virus and cytomegalovirus, all of which were negative.

Moreover, a basic metabolic evaluation was performed at this point for amino acid disorders (including tyrosinemia type I), galactosemia, and mitochondrial disorders, which was unremarkable. This included measurements of blood ammonia and lactate, plasma amino acid chromatography, newborn screening by tandem mass spectrometry, and assessment of galactose-1-phosphate uridyltransferase activity.

The combination of parental consanguinity and a maternal cluster of end-stage liver disease (two transplanted relatives) heightened concern for autosomal-recessive hepatopathies, such as Wilson disease, and other genetic cholestatic disorders that frequently present in childhood and may progress to cirrhosis and transplant.

At the same time, autoimmune hepatitis remained a leading consideration; therefore, total immunoglobulin G was taken and was found to be elevated at 2445 mg/dL (normal for age: 540–1820 mg/dL). Further autoimmune hepatitis panel (antinuclear antibodies, anti-smooth muscle antibody, anti-liver kidney microsomal type 1, and anti-soluble liver antigen), liver biopsy, and exome sequencing were requested.

In the context of the local healthcare setting and financial limitations, the patient’s family chose not to pursue liver biopsy due concerns about its invasiveness and the possibility of inconclusive histopathological findings; they opted instead to proceed solely with genetic testing. Meanwhile, the patient developed severe pneumonia with respiratory distress requiring artificial ventilation in another hospital. He passed away a few days later from sepsis.

Exome sequencing using next-generation sequencing (performed at Centogene- GmbH, a commercial Clinical Laboratory Improvement Amendments certified laboratory) identified a novel homozygous pathogenic variant in the *AIRE* gene, NM_000383.4: c.1066dup (p.Arg356fs), which creates a shift in the reading frame starting at codon 356 in exon no. 9 (of 14), confirming the diagnosis of autosomal recessive APECED.

Endocrine evaluation including parathyroid and thyroid functions, adrenal axis, and glucose metabolism was negative (Table 1).

## 3. Discussion

The clinical diagnosis of APECED is traditionally based on the classic triad of hypoparathyroidism, adrenal insufficiency, and chronic mucocutaneous candidiasis. The diagnosis is confirmed by genetic testing demonstrating a pathogenic variant in the AIRE gene [10]. Redefined criteria were described by Ferre et al. in 2016, adding urticarial eruption, intestinal dysfunction, and enamel hypoplasia to the classic triad [11]. Using the Ferre–Lionakis criteria, our patient presented with one symptom from the classic triad, chronic mucocutaneous candidiasis, as the initial clinical manifestation, accompanied by a non-classic feature (enamel hypoplasia), and was found to carry a pathogenic *AIRE* variant [6,11]. Additional clinical findings included recurrent keratitis (partly attributed to exposure keratitis resulting from craniosynostosis and partly to filamentary keratitis), diffuse hair thinning across the scalp, and short stature associated with markedly low insulin-like growth factor-1 level.

At presentation, the primary objective was to determine the etiology of the hepatitis in the context of a strong family history of liver failure. The presence of consanguinity and two closely related individuals with liver failure requiring liver transplantation strongly suggested a genetic etiology of the liver disease. Details of their medical files were not available, as they were not followed in our medical center.

Given the extensive negative infectious and metabolic workup, the identification of a novel homozygous loss-of-function *AIRE* variant provides strong support for a diagnosis of APECED, even though autoimmune hepatitis-associated serologies (including APECED-related hepatic autoantibodies) could not be assessed due to financial and clinical limitations.

Autoimmune hepatitis has been reported to occur in approximately 10% of APECED patients from European populations, with significantly higher prevalence (42%) in American cohorts [9,11]. To the best of our knowledge, no prevalence data is available from the Middle East countries. As described by Chascsa et al., hepatitis may present early in the course of the disease before classic manifestations of APECED in 33% of cases of a cohort of 18 patients. In addition, the standard autoimmune hepatitis-associated biomarkers were positive in a minority of patients [9].

In another American cohort of 35 patients with APECED [11], associated hepatitis was uncommon in early childhood but increased with age in the pediatric range, occurring in approximately 9% of patients by age 2, about 11% by age 5, and rising to around 23% by age 10. The prolonged use of intermittent systemic steroids in our patient, initially prescribed by his primary care physician for alopecia, may have delayed the progression of hepatitis.

The novel homozygous *AIRE* variant identified in this patient has been described using standardized Human Genome Variation Society (HGVS) nomenclature as NM_000383.4:c.1066dup, p.(Arg356Profs*16), reflecting a single-nucleotide duplication predicted to introduce a frameshift and premature termination codon. It is classified as likely pathogenic or pathogenic, according to ClinVar [12]. Notably, this variant has not been previously reported in individuals with *AIRE*-related disorders and is absent from population databases. The identified sequence change creates a premature translational stop signal (p.Arg356Profs*16) in the *AIRE* gene, likely leading to an absent or disrupted protein product. Loss-of-function variants in *AIRE* gene [12,13,14], with numerous frameshift and nonsense variants, are reported as pathogenic in ClinVar across diverse populations.

The range of *AIRE* gene variants differs between populations. The most prevalent pathogenic variant in Finnish populations is c.769C>T (p.Arg257Ter), which is frequently called the “Finnish major mutation.” Numerous reports have found that this pathogenic variant is common and linked to the traditional presentation of APECED [4,15,16]. The genetic landscape of *AIRE* pathogenic variants is more varied in North American populations, especially in the United States of America. One of the most prevalent pathogenic variants seen in American patients, who frequently exhibit a wider variety of non-endocrine symptoms (including hepatitis) than their European counterparts, is the c.967_979del13 pathogenic variant [11]. Recent studies suggest that phenotypic outcomes can vary markedly due to the influence of genetic background, epigenetic regulation, and environmental factors [17].

The inference of pathogenicity for this novel variant NM_000383.4: c.1066dup, p.(Arg356Profs*16) is based entirely on in silico prediction and established biological knowledge of *AIRE*, rather than on direct functional assays. Moreover, it is not possible to attribute the early-onset and severe hepatic phenotype to this variant from a single case in the absence of functional validation or comparative genotype–phenotype data.

Next-generation sequencing did not reveal any clinically relevant variants associated with craniosynostosis, suggesting that the craniosynostosis observed in this patient represents an incidental and unrelated comorbidity.

This case underscores the importance of incorporating the Ferre–Lionakis criteria, alongside the classic triad for the accurate diagnosis of APECED. It also highlights the necessity of including APECED in the differential diagnosis when evaluating cases of unexplained hepatitis, particularly in patients with a suggestive clinical or family history. Furthermore, the early implementation of next-generation sequencing in the diagnostic workup can significantly expedite diagnosis and facilitate the timely initiation of targeted therapies, potentially improving patient outcomes [18].

Further studies are warranted to investigate this novel *AIRE* pathogenic variant, particularly to assess its potential association with hepatic manifestations in APECED. Broader prevalence studies could also elucidate the significance and frequency of this pathogenic variant in affected populations, thereby improving our understanding of its clinical implications.

## Figures and Tables

**Table 1:** Endocrine evaluation of a patient with Autoimmune Polyendocrinopathy with Candidiasis and Ectodermal Dystrophy.

Test	Result	Reference Range
Calcium	8.6	8.5–10.5 mg/dL
Phosphate	4.2	3.2–5.7 mg/dL
Parathyroid hormone	23.3	15–76 pg/mL
TSH	1.010	0.700–5.970 µU/mL
Free T4	1.50	0.96–2.1 ng/dL
Morning cortisol	7.2	3.7–19.4 µg/dL
Glucose	93	76–110 mg/dL
ACTH (adrenocorticotropic hormone)	Not done	
ACTH stimulation test	Not done	
HBA1c	4.5%	below 5.7%
Sodium	138	135–145 mmol/L
Potassium	3.7	3.5–5.1 mmol/L
Chloride	102	98–109 mmol/L
Carbon dioxide	23	24–30 mmol/L
17-hydroxyprogesterone	9.4	0–30 nmol/L

## Data Availability

The original contributions presented in this study are included in the article. Further inquiries can be directed to the corresponding author.

## Abbreviations

The following abbreviations are used in this manuscript:

APECED	Multidisciplinary Digital Publishing Institute
*AIRE*	Autoimmune regulator
ALT	Alanine aminotransferase
AST	aspartate aminotransferase
